# Construction of *Polygonatum sibiricum* Polysaccharide Functionalized Selenium Nanoparticles for the Enhancement of Stability and Antioxidant Activity

**DOI:** 10.3390/antiox11020240

**Published:** 2022-01-26

**Authors:** Wanwen Chen, Hao Cheng, Wenshui Xia

**Affiliations:** 1 State Key Laboratory of Food Science and Technology, School of Food Science and Technology, Jiangnan University, Wuxi 214122, China; wwchen@jiangnan.edu.cn (W.C.); xiaws@jiangnan.edu.cn (W.X.); 2 Collaborative Innovation Center of Food Safety and Quality Control in Jiangsu Province, Jiangnan University, Wuxi 214122, China

**Keywords:** selenium nanoparticles, *Polygonatum sibiricum* polysaccharide, stability, antioxidant

## Abstract

Although selenium nanoparticles (SeNPs) have attracted great attention due to their potential antioxidant activity and low toxicity, the application of SeNPs is still restricted by their poor stability. A combination of polysaccharides and SeNPs is an effective strategy to overcome the limitations. In this study, *Polygonatum sibiricum* polysaccharide (PSP) was used as a stabilizer to fabricate SeNPs under a simple redox system. Dynamic light scattering, transmission electron microscopy, energy dispersive X-ray, ultraviolet-visible spectroscopy, Fourier transform infrared, and X-ray photoelectron spectrometer were applied to characterize the synthesized PSP-SeNPs. The stability and the antioxidant activity of PSP-SeNPs were also investigated. The results revealed that the zero-valent and well-dispersed spherical PSP-SeNPs with an average size of 105 nm and a negative ζ-potential of −34.9 mV were successfully synthesized using 0.1 mg/mL PSP as a stabilizer. The prepared PSP-SeNPs were stable for 30 days at 4 °C. The decoration of the nanoparticle surface with PSP significantly improved the free radical scavenging ability of SeNPs. Compared to the H_2_O_2_-induced oxidative stress model group, the viability of PC-12 cells pretreated with 20 μg/mL PSP-SeNPs increased from 56% to 98%. Moreover, PSP-SeNPs exhibited a higher protective effect on the H_2_O_2_-induced oxidative damage on PC-12 cells and lower cytotoxicity than sodium selenite and SeNPs. In summary, these results suggest the great potential of PSP-SeNPs as a novel antioxidant agent in the food or nutraceuticals area.

## 1. Introduction

Selenium is an essential micronutrient for humans and animals [1]. It is an integral component of more than 30 kinds of selenoproteins and selenium-containing enzymes, such as selenoprotein P (SelP), selenoprotein S (SelS), selenoprotein M (SelM), subfamilies of thioredoxin reductases (TrxR), glutathione peroxidases (GPx), and iodothyronine deiodinases (ID), that play a key role in regulating redox balance and preventing cellular damage from radicals [2,3]. However, at least one billion people in the world are at risk of selenium deficiency at present because the intake of selenium is insufficient to meet the daily requirement [4]. Epidemiological studies established that selenium deficiency is associated with many diseases, including premature aging, a decline in sperm motility, myocardial failure, neurological diseases, endemic osteoarthropathy (Keshan disease), and ischemic heart disease [5]. Although high-dose sodium selenite, methyl selenium, and selenocysteine exhibit excellent bioactivities, they can also result in serious toxicity problems, leading to many diseases [6]. Thus, it is of great importance to seek novel selenium species as food supplements or additives.

Selenium nanoparticles (SeNPs) have gained much attention owing to their unique physical, chemical, and antioxidant activities [7]. Moreover, SeNPs have higher bioavailability and lower toxicity in comparison to other chemical forms of selenium, making them the promising alternative selenium source in food dietary [8]. However, SeNPs alone with valence state zero are highly unstable in an aqueous solution and easily transform to aggregate, resulting in lower bioactivity and further limiting their practical application [9]. Many efforts have been made to develop a simple, efficient, and green strategy for the dispersion and stabilization of SeNPs using bioactive templates [10]. Natural polysaccharides not only have complex structures, large specific surface areas, and ionizable functional groups but also possess excellent biocompatibility and biodegradability [11]. These features could decrease the surface energy of SeNPs, further preventing aggregation through electrostatic interaction or hydrogen bonds. Thus, polysaccharides applied as carriers to fabricate SeNPs with desired characteristics, such as stability and functionality, using the green chemical method is drawing much attention recently. For example, numerous studies reported that chitosan (CS) could be used as templates to prepare uniform SeNPs and the ligated SeNPs remain stable for over 1 month [12]. However, the superior properties of CS are limited due to its water insolubility and our previous research also found that CS-SeNPs aggregated under alkaline conditions (pH ≥ 9) [13]. Several polysaccharides derived from fungi [14], fruit [15], and medicinal plants [16] have been demonstrated to enhance the antioxidant activity of SeNPs. Recently, medicinal plant polysaccharides have attracted increasing attention due to their significant bioactivities with no side effects [17]. Therefore, it can be expected that the combination of medicinal plant polysaccharides with SeNPs will reduce the inherent limitations and enhance the benefits of selenium and polysaccharides.

*Polygonatum sibiricum* is a traditional Chinese herbal medicine, belonging to the *Liliaceae* family, which has been introduced in the 2015 edition of pharmacopeia [18]. China has abundant resources of *Polygonatum sibiricum*, especially in the south of the Yangtze River [19]. The constituents of *P. sibiricum* include polysaccharides, saponins, flavonoids, alkaloids, lignin, vitamins, and a variety of trace elements, of which polysaccharides are the major pharmacologically active ingredients [20]. In the last three years, *Polygonatum sibiricum* polysaccharides (PSP) are demonstrated to exhibit a wide range of pharmacological activity [21], such as osteogenic activity [22], anti-diabetes [23], immunological activity [24], and especially antioxidant activity, which makes them suitable for application in functional foods and therapeutic agents. PSP demonstrated strong antioxidant properties, which could attenuate D-gal-induced heart aging [25] and protect the mice livers against ethanol-induced oxidative damage via inhibiting oxidative stress [26]. However, no study has been reported using PSP as a decorator to functionalize SeNPs.

In this study, considering the antioxidant activity of PSP as well as the drawbacks of SeNPs, a combined strategy was conducted to fabricate SeNPs using PSP as a stabilizer in the redox system of sodium selenite (Na_2_SeO_3_) and ascorbic acid (Vc) through a simple chemistry approach. The synthesized PSP functionalized SeNPs (PSP-SeNPs) were characterized by dynamic light scattering (DLS), transmission electron microscopy (TEM), energy dispersive X-ray (EDX), ultraviolet-visible spectroscopy (UV-vis), Fourier transform infrared (FTIR), and X-ray photoelectron spectrometer (XPS). The physicochemical stabilities of synthesized nanoparticles under varying conditions, including ionic strength, pH, and temperature, were analyzed. In addition, the antioxidant activity of PSP and PSP-SeNPs was quantified by ABTS and DDPH free radical scavenging assays. Moreover, the protective effect on the H_2_O_2_-induced cell death was also investigated by MTT assay.

## 2. Materials and Methods

### 2.1. Reagents

Commercial *Polygonatum sibiricum* polysaccharide (PSP) with a purity of 95% and a molecular weight of 14 kDa was obtained from Qiannuo Biotechnology Co. Ltd. (Xi’an, China), sodium selenite (Na_2_SeO_3_), hydrogen peroxide (H_2_O_2_), ascorbic acid (Vc), potassium persulfate (K_2_S_2_O_8_), 1, 1-diphenyl-2-picrylhydrazyl (DPPH), 2, 2-azinobis (3-ethylbenzothiazoline-6-sulfonic acid) and diammonium salt (ABTS) were purchased from Sinopharm Chemical Reagent Co., Ltd. (Shanghai, China). All chemicals used were of analytical grade, and the water used in all experiments was obtained from the Milli-Q system.

### 2.2. Preparation of SeNPs and PSP Stabilized SeNPs

PSP-SeNPs were prepared according to the procedure described by Ye et al. with minor modification [8]. PSP stock solution (5 mg/mL) was freshly prepared. Where 1 mL of sodium selenite solution (50 mM) was mixed with various volumes of PSP solution under stirring for 5 min. Then 1 mL of ascorbic acid solution (200 mM) was added dropwise into the mixture, and it was reconstituted to a final volume of 10 mL with Milli-Q water. The reaction was carried out at room temperature for 30 min. Finally, the solution was dialyzed using regenerated cellulose tubes (Mw cutoff 3500 Da) against ultrapure water for 48 h at 4 °C. The final concentrations of PSP were 0.01, 0.05, 0.075, 0.1, 0.125, 0.15, 0.25 mg/mL. SeNPs were synthesized in the absence of PSP through the same procedure as above. The resultant products were lyophilized to obtain the freeze-dried nanocomposites. The concentration of selenium was determined by the Optima 8300 inductively coupled plasma optical emission spectrometer (ICP-OES, PerkinElmer, Billerica, MA, USA).

### 2.3. Characterization

The mean diameter, size distribution, and ζ-potential of nanocomposites were determined using a Zetasizer Nano ZS analyzer (Malvern Instruments Corporation, Worcestershire, UK). The morphology was observed using transmission electron microscopy (TEM) (JEOL, JEM-2100, Tokyo, Japan). Samples for TEM observation were prepared by placing one drop of SeNPs and PSP-SeNPs aqueous solution on a carbon-coated copper grid and dried at room temperature. The micrographs were acquired at the accelerating voltage of 200 kV. The elemental composition and distribution were determined by the energy dispersive X-ray (EDX) analysis performed on a high-resolution transmission electron microscopy (HRTEM) (JEOL, JEM-2100, Tokyo, Japan). The ultraviolet-visible (UV-vis) spectrophotometer (UV-1800, Shimadzu Corporation, Tokyo, Japan) was used to measure the UV-vis absorption spectra of SeNPs and PSP-SeNPs solutions in the wavelength range of 190–800 nm with an interval of 1.0 nm. The Fourier transform infrared (FTIR) spectra were recorded on a Nicolet iS 10 instrument (Thermo Fisher Scientific, Waltham, MA, USA). Each sample was grounded with KBr, pressed into uniform pellets, and scanned in the wavenumber range of 4000–400 cm^−^^1^ with a resolution of 4.0 cm^−^^1^ using pure KBr as the background. The X-ray photoelectron spectrometer (XPS) was used to analyze the valence states of the elements. The XPS patterns were operated on a Thermo Scientific ESCALab 250Xi+ (Thermo Fisher Scientific, Waltham, MA, USA) using 150 W monochromated Al Kα radiation.

### 2.4. Stability of PSP-SeNPs

The stability of PSP-SeNPs under various conditions was investigated according to the methods described previously [27]. To determine the effect of ionic concentration on stability, 10 mL of PSP-SeNPs were mixed with different concentrations of NaCl solution (10, 50, and 100 mM). The effect of pH on the stability of NPs was analyzed by adjusting the pH of PSP-SeNPs to 2, 3, 4, 5, 6, 7, 8, 9, and 10 using 0.1 M HCl or NaOH. Where 10 mL of PSP-SeNPs were incubated in a water bath at different temperatures (25, 50, 70, and 90 °C) to investigate the effect of temperature on the stability of PSP-SeNPs. After being stabilized for 1 h, their mean diameter and ζ-potential were determined using a Zetasizer Nano ZS analyzer. In addition, PSP-SeNPs solutions were stored at 4 °C for 30 days to investigate the short-term storage stability by determining the mean diameter and ζ-potential.

### 2.5. Antioxidant Assays

#### 2.5.1. DPPH Radical Scavenging Ability

The DPPH radical scavenging activity was determined referring to the methods reported previously with minor modifications [14]. Various concentrations of PSP, SeNPs, PSP-SeNPs, and Vc at 0.01, 0.05, 0.1, 0.25, 0.5, 0.75, 1.0 mg/mL were prepared. Further, 2 mL of the sample solutions were mixed with an equal volume of freshly prepared DPPH solution (50 mg/L) in ethanol. The mixture was shaken vigorously and incubated in darkness at 33 °C for 30 min. The absorbance was measured at 517 nm using a UV-vis spectrophotometer. Vc was used as a positive control. The scavenging activity was calculated as follows:

(1) $$\mathrm{DPPH}\;\mathrm{radical}\;\mathrm{scavenging}\;\mathrm{ability}\;\left(\%\right)=\left(1\;-\;\frac{A_{a}\;{-\;A}_{b}}{A_{c}}\right)\;\times \;100$$

where A_a_ is the absorbance of the sample mixed with DPPH solution, A_b_ is the absorbance of the sample in the absence of the DPPH solution, A_c_ is the absorbance of the DPPH solution without the sample as a blank control.

#### 2.5.2. ABTS Radical Cation Decolonization Assay

The assay of ABTS radical cation scavenging ability was performed as described previously with some modification [28]. ABTS and potassium persulfate (K_2_S_2_O_8_) were dissolved in distilled water. A stock solution of ABTS^•+^ was prepared by mixing 7.4 mM ABTS solution with 2.6 mM K_2_S_2_O_8_ solution. The mixture was incubated for 12 h in the dark to reach equilibrium. The ABTS^•+^ stock solution was diluted with sodium phosphate buffer (pH 7.4) to obtain an optical density of 0.70 ± 0.02 at 734 nm. Then 1 mL of different concentrations of PSP, SeNPs, PSP-SeNPs, and Vc (0.01, 0.05, 0.1, 0.25, 0.5, 0.75, 1.0 mg/mL) was added to 4 mL of diluted ABTS^•+^ solution. The mixture was vigorously blended and incubated at room temperature for 6 min in darkness. The absorbance was measured at 734 nm using a UV-vis spectrophotometer. The ability to scavenge ABTS^•+^ was calculated by Equation (2).

(2) $${\mathrm{ABTS}}^{\text{•}+}\mathrm{radical}\;\mathrm{scavenging}\;\mathrm{ability}\;\left(\%\right)=\left(1\;-\;\frac{A_{d}\;{-\;A}_{e}}{A_{f}}\right)\;\times \;100\;$$

where A_d_ is the absorbance of the sample mixed with the ABTS^•+^ solution, A_e_ is the absorbance of the sample in the absence of the ABTS^•+^ solution, A_f_ is the absorbance of the ABTS^•+^ solution without the sample.

### 2.6. Cells Culture and MTT Assays

PC-12 cells were cultured in Dulbecco’s modified Eagle’s medium (DMEM) supplemented with 10% fetal bovine serum (FBS) and 1% antibiotic mixture (100 U/mL penicillin and 100 μg/mL streptomycin). The cytotoxic effects of different selenium concentrations of PSP-SeNPs, SeNPs, and Na_2_SeO_3_ on cells were tested using MTT assays [15]. Cells were seeded in a 96-well plate at a density of 1 × 10^4^ cells/well and incubated at 37 °C in a CO_2_ incubator (5% CO_2_ and 95% relative humidity) for 24 h. Then the medium was removed and cells were treated with different concentrations of samples prepared in DMEM with 10% FBS for an additional 24 h. After incubation, 20 μL of MTT (5 mg/mL) was added to each well and incubated at 37 °C for 3 h. Then the supernatant was removed and 150 μL of DMSO was added. The absorbance was measured by a microplate reader at 570 nm. The cell viability was calculated by Equation (3).

(3) $$\mathrm{Cell}\;\mathrm{viability}\;\left(\%\right){=\mathrm{OD}}_{\mathrm{sample}}{/\mathrm{OD}}_{\mathrm{control}\;}\times \;100\;$$

where OD_sample_ is the absorbance of the treated cells and OD_control_ is the absorbance of the control cells.

To determine the protective effect of PSP-SeNPs, SeNPs, and Na_2_SeO_3_ on H_2_O_2_-induced cell cytotoxicity, cells were pre-incubated with different selenium concentrations of samples prepared in DMEM with 10% FBS for 24 h. After incubation, the medium was removed and cells were washed with PBS. Then cells were treated with a medium containing 500 μM H_2_O_2_ for 12 h. The medium was removed and the cell viability was determined by MTT assay as described above.

### 2.7. Statistical Analysis

All the experiments were performed at least in triplicate. The results were expressed as mean ± standard deviation (SD). Statistical analysis was carried out using paired t-tests for comparing means of two samples by the SPSS 20.0 statistical software (IBM, Armonk, NY, USA). Statistical differences between samples were performed with a level of significance of *p* < 0.05.

## 3. Results

### 3.1. The Synthesis of SeNPs and PSP-SeNPs

**Figure 1:**
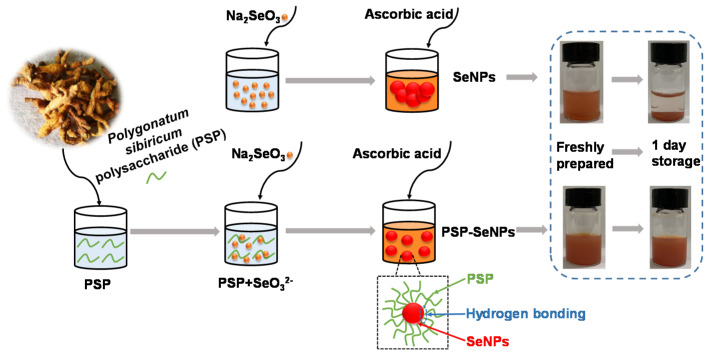
Synthetic scheme for the preparation of selenium nanoparticles (SeNPs) and Polygonatum sibiricum polysaccharide stabilized selenium nanoparticles (PSP-SeNPs) and images of the dispersions before and after storage for 1 day.

In the present study, SeNPs and PSP-SeNPs were prepared using a simple redox system of ascorbic acid and sodium selenite in the absence and presence of PSP as the stabilizer and capping agent. The visual color of the reaction solution is an indicator to preliminary infer the formation of selenium nanoparticles [29]. As shown in Figure 1, the red color of the solution indicated the SeO_3_^2−^ was successfully reduced to either monoclinic or amorphous SeNPs [16]. In addition, the SeNPs in the presence of PSP showed a uniform red color and were stable in the aqueous solution. However, SeNPs without the decoration of PSP aggregated into precipitation after 1 day of storage, whereas no significant changes were observed in the solution of PSP-SeNPs. This might be attributed to the high surface energy, leading to the aggregation of SeNPs [9]. Hence, PSP plays a key role in the formation and stabilization of SeNPs.

### 3.2. The Size and ζ-Potential Analysis of SeNPs and PSP-SeNPs

**Figure 2:**
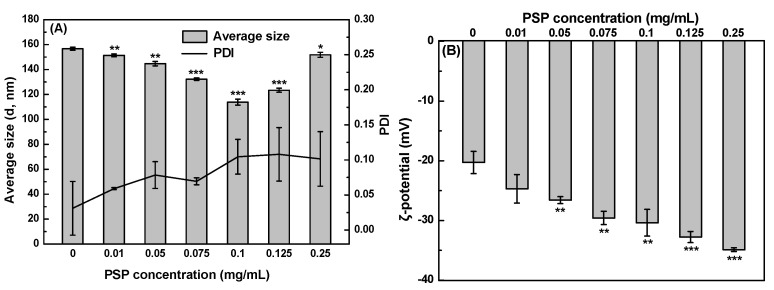
Size distribution (**A**) and ζ-potential (**B**) of SeNPs and PSP-SeNPs prepared with different concentrations of PSP (0.01–0.25 mg/mL). Values marked with *: *p* < 0.05, **: *p* < 0.01, and ***: *p* < 0.001 indicated significant differences when compared to SeNPs.

The concentration of the polysaccharides is an important factor that influences the size of SeNPs, further affecting their functionality in food or medical application [30]. Thus, the effect of PSP concentrations on the hydrated particle size and the corresponding polydispersity index (PDI), as well as the ζ-potential of nanoparticles in the aqueous solution was investigated first. The particle size of barely SeNPs was up to 157 nm (Figure 2A). The addition of PSP at different concentrations could decrease the average size of SeNPs. The average diameter of PSP-SeNPs significantly decreased from 151 to 132 nm as the concentration of PSP increased from 0.01 to 0.075 mg/mL. PSP-SeNPs showed the smallest average size of 114 nm at the PSP concentration of 0.1 mg/mL, whereas further increases in PSP concentration from 0.125 to 0.25 mg/mL resulted in an increase in the size from 123 to 152 nm. It might be due to PSP at a low concentration was not enough to control the formation of SeNPs and prevent them from aggregation [31]. On the other hand, too high PSP concentration represented more PSP chains coated on the surface of SeNPs, resulting in a larger hydration particle size [32]. As shown in Figure 2B, SeNPs in the absence of PSP exhibited a negative ζ-potential at −20.3 mV. The ζ-potential values of PSP-SeNPs were determined to be approximately −24.7, −26.6, −29.6, −30.4, −32.8, −34.9 mV at the PSP concentration of 0.01, 0.05, 0.075, 0.1, 0.125, 0.25 mg/mL. The absolute ζ-potential values of PSP-SeNPs increased with the PSP concentration increasing, further demonstrating that negatively charged PSP was exposed on the surface of SeNPs. Moreover, the higher magnitude of ζ-potential represents greater stability of nanoparticles [13], suggesting that the SeNPs decorated with PSP possess higher stability than barely SeNPs. PSP-SeNPs prepared by 0.1 mg/mL PSP were used in the following experiments.

### 3.3. Morphological and Structural Characterizations of SeNPs and PSP-SeNPs

**Figure 3:**
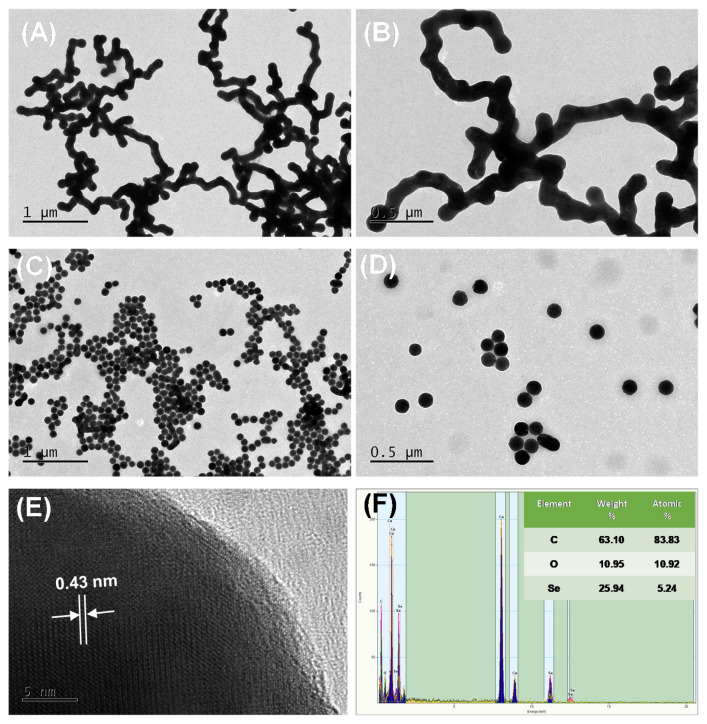
TEM images of SeNPs (**A**,**B**) and PSP-SeNPs in the presence of 0.1 mg/mL PSP (**C**,**D**). HRTEM of an individual PSP-SeNPs (**E**) and typical EDX from HRTEM (**F**).

The morphology and size of SeNPs and PSP-SeNPs were further characterized by TEM. Figure 3A,B exhibited the TEM images of SeNPs in the absence of PSP. The results showed that adjacent SeNPs agglomerated together and presented a dendritic structure. The large-sized cluster and aggregates can also be easily visualized. However, the SeNPs in the presence of 0.1 mg/mL PSP (Figure 3C,D) exhibited a homogeneous and monodisperse spherical structure with an average size of about 105 nm, confirming the important role of PSP in regulating and stabilizing SeNPs. It should be pointed out that the hydrodynamic radius of the nanoparticles provided in the DLS analysis was larger than the size observed in the TEM image, which was sensitive to the electron-rich nanoparticles. The HRTEM image (Figure 3E) of an individual PSP-SeNPs showed a distinct lattice fringe with an interplanar spacing of 0.43 nm, revealing the excellent crystallinity of PSP-SeNPs. The elemental composition and distribution of the PSP-SeNPs were further determined by EDX. As shown in Figure 3F, the strong C, O, and Se element peaks were observed in EDX spectra. The PSP-SeNPs had a 63.10% weight percentage of C atom, together with 10.95% O atom and 25.94% Se atom. Furthermore, no other peaks for other elements were detected, indicating that PSP was successfully coated on the surface of SeNPs and confirming the purity of PSP-SeNPs [33].

### 3.4. The Stability of SeNPs and PSP-SeNPs

**Figure 4:**
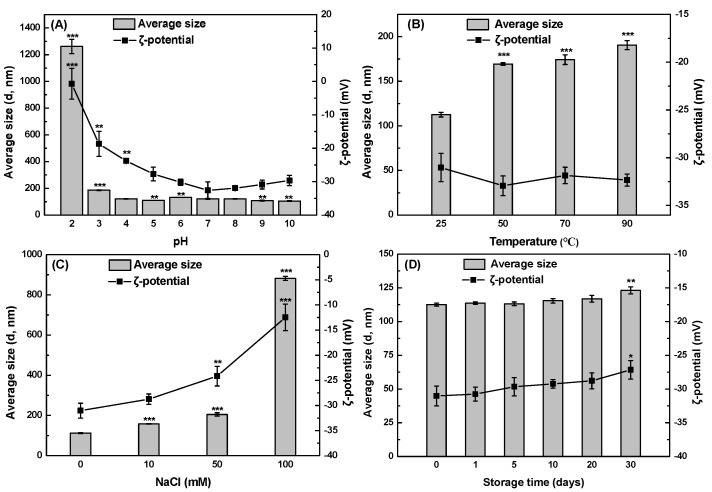
Effect of pH (**A**), temperature (**B**), ion strength (**C**), and storage time (**D**) on the average size and ζ-potential of PSP-SeNPs. Values marked with *: *p* < 0.05, **: *p* < 0.01, and ***: *p* < 0.001 indicated significant differences when compared to the conditions of pH: 7, temperature: 25 °C, NaCl: 0 mM, or storage time: 0 day.

Stability is an important factor influencing the functionality and applications of nanomaterials. In this study, the effect of pH, temperature, and ionic strength on the stability of PSP-SeNPs was investigated. As shown in Figure 4A, the average size of PSP-SeNPs significantly decreased from 1262 to 186 nm when pH was increased from 2 to 3. It could be observed that no obvious changes occurred in the average size at pH range from 4 to 10. Similar results were also described previously on the stability of Polyporus umbellatus polysaccharide (PUP) coated SeNPs [34]. This might be ascribed to the protonation of PSP at pH 2 that weakened the electrostatic interactions between SeNPs and PSP, leading to the aggregation of nanoparticles. Moreover, the ζ-potential of PSP-SeNPs kept increasing with pH increased and reached the highest value of −32.6 mV at pH 7. A further increase in pH did not significantly affect the ζ-potential of PSP. It has been reported that the ζ-potential of nanoparticles was highly associated with the pKa value of the polysaccharides. The pH value higher than the pKa of polysaccharides resulted in more deprotonated characteristic groups, contributing to the increase in ζ-potential [27]. The average size of PSP-SeNPs increased from 113 to 191 nm, accompanied by the temperature increase from 25 °C to 90 °C with a constant ζ-potential at around −31 mV (Figure 4B). The result indicated that heating could increase the chances and strength of collisions, resulting in a larger size [29]. As shown in Figure 4C, the particle size of PSP-SeNPs exhibited a slight increase in 10 and 50 mM NaCl with decreased ζ-potential, and steeply increased to 882 nm in a high concentration of NaCl at 100 mM. High ion strength could remarkably reduce the surface charge of nanoparticles due to the electrostatic interaction between positive charged Na^+^ and negatively charged PSP-SeNPs, resulting in the decrease of the electrostatic repulsion among nanoparticles [35]. It was observed that PSP-SeNPs were stable at about 113 nm for at least 20 days of storage (Figure 4D). The stability of PSP-SeNPs was higher than that of SeNPs decorated with a hyperbranched polysaccharide from Lignosus rhinocerotis 14. It should be pointed out that SeNPs in the absence of PSP precipitated after 1-day storage (Figure 1). Moreover, the particle size of PSP-SeNPs only increased from 113 to 123 nm after 30 days of storage and the ζ-potential of PSP-SeNPs presented at around −30 mV during the storage time, suggesting that PSP-SeNPs had better stability.

### 3.5. Characterization and Possible Stabilizing Mechanism of PSP-SeNPs

**Figure 5:**
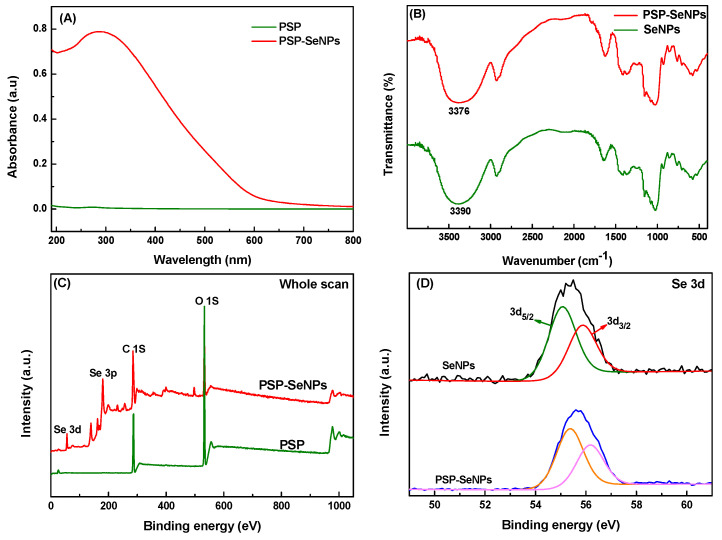
UV-vis spectra (**A**), FTIR spectra (**B**), XPS spectra (**C**), and XPS spectra of Se 3d (**D**) of PSP and PSP-SeNPs.

The UV-vis spectra of PSP and PSP-SeNPs in the range of 190 to 800 nm were presented in Figure 5A. It was shown that no characteristic absorption peaks were observed on the UV-vis spectra of PSP at the concentration of 0.01 mg/mL. The PSP-SeNPs exhibited wide absorption bands with a maximum absorption peak at about 288 nm. The characteristic absorption peak corresponded to a localized surface plasmon response (LSPR), further demonstrating the formation of nanoparticles [36].

FTIR spectra were performed to clarify the interaction between PSP and SeNPs. In the spectrum of PSP (Figure 5B), the broad absorption band at nearly 3390 cm^−1^ was assigned to the O-H stretching vibration. The peak presented at 2927 cm^−1^ was attributed to the C-H stretching vibration. The signals that occurred in the region of 1200–1000 cm^−1^ were associated with the C-O stretching vibration, indicating the existence of a pyranose ring [37]. The FTIR spectrum of PSP-SeNPs was similar to that of the pure PSP, indicating the presence of PSP on the surface of SeNPs. In addition, the O-H stretching vibration occurred red-shift from 3390 cm^−1^ to 3376 cm^−1^, suggesting the formation of hydrogen bonds between SeNPs and the PSP chains [38]. Based on the above results, we proposed that the interaction mechanism was similar to the combination of arabinogalactans/and SeNPs as described previously [36]. Briefly, the SeO_3_^2−^ reacted with the -OH group in the PSP molecule to form special chain-shaped intermediates first, then reduced to the element Se by ascorbic acid. The Se atom further aggregated into the nucleus to form SeNPs as the reaction processed and the -OH groups of PSP were bound to the surface of SeNPs to prevent the aggregation of nanoparticles.

The XPS spectra were further used to analyze the valence state of selenium. The peaks of Se 3d and 3p orbitals at the binding energy of 55.6 and 179.3 eV (Figure 5C) indicated the zero-valent state of Se within the PSP-SeNPs [10]. As shown in Figure 5D, the peaks of Se 3d_5/2_ and Se 3d_3/2_ were up-shifted from 55.1 and 55.9 (SeNPs) to 55.4 and 56.2 (PSP-SeNPs), respectively. The results indicate that the Se 3d orbit participated in the formation of PSP-SeNPs [39], confirming that PSP was successfully conjugated to the SeNPs. Meanwhile, no peak was found at 59.5 eV, which represented the typical Se 3d signal of Se (IV), suggesting that Se (IV) was completely reduced to elemental selenium [40].

### 3.6. Antioxidant Assays

**Figure 6:**
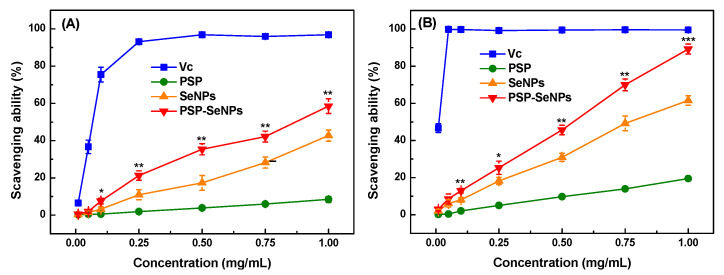
Antioxidant activities of PSP, SeNPs, and PSP-SeNPs in vitro. (**A**) DPPH radical scavenging activity. (**B**) ABTS radical scavenging activity. Ascorbic acid (Vc) is used as a positive control. Values marked with *: *p* < 0.05, **: *p* < 0.01, and ***: *p* < 0.001 indicated significant differences when compared to SeNPs at the same concentration.

The DPPH and ABTS radical scavenging activity were measured in our study to evaluate the antioxidant activity of PSP, SeNPs, and PSP-SeNPs. As shown in Figure 6A, PSP exhibited a low DPPH radical scavenging ability at the tested concentrations. Both SeNPs and PSP-SeNPs had a concentration-dependent DPPH radical scavenging effect at 0.01–1.0 mg/mL. PSP-SeNPs showed a higher scavenging ability than SeNPs. The scavenging effect of PSP-SeNPs reached 59% at the concentration of 1.0 mg/mL, whereas SeNPs could only scavenge 43% DPPH radical at the same concentration. This might be attributed to the enhanced hydrogen-donating ability of PSP-SeNPs to form a stable DPPH-H molecule [41]. Compared to the DPPH radical, all the tested samples performed more efficiently in scavenging ABTS radical (Figure 6B). Similar to the DPPH scavenging assay, the ABTS radical scavenging capacity of PSP-SeNPs was significantly stronger than that of PSP and SeNPs. At 1.0 mg/mL, the scavenging effects of PSP, SeNPs, and PSP-SeNPs were 20%, 62% and 89%, respectively. It has been reported that the DPPH scavenging ability of gum arabic-selenium nanocomposites was lower than 60% at 1.0 mg/mL [42]. The ABTS radical scavenging activity of SeNPs functionalized with a polysaccharide from *Rosa roxburghii* fruit only reached about 50% at 1.0 mg/mL 15. The free radical scavenging ability of PSP-SeNPs synthesized in our study was higher than the above nanoparticles. Moreover, the results showed that the surface decoration of SeNPs with PSP could remarkably improve the antioxidant activity of SeNPs and PSP. PSP-SeNPs with a smaller size could provide more radical reactive sites due to their larger specific surface area, resulting in higher antioxidant activity [29,43]. However, barely SeNPs were easily aggregated with a decreased active surface to react with the free radicals, further reducing their biological activities [43].

### 3.7. Effects of PSP-SeNPs on H_2_O_2_-Induced PC-12 Cells Toxicity

**Figure 7:**
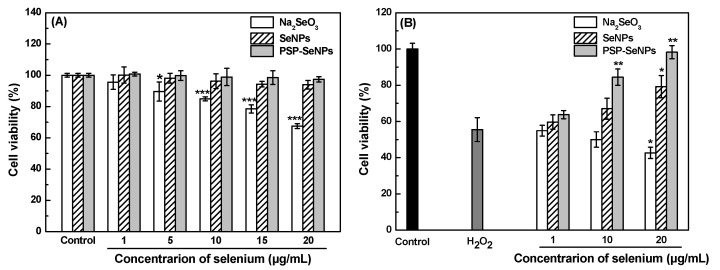
Effects of sodium selenite (Na_2_SeO_3_), SeNPs, and PSP-SeNPs on the viability of PC-12 cells (**A**). Values marked with *: *p* < 0.05 and ***: *p* < 0.001 indicated significant differences when compared to the control group. The protective effect against H_2_O_2_ (0.5 µM)-induced PC-12 cells toxicity by MTT assay (**B**). Values marked with *: *p* < 0.05 and **: *p* < 0.01 indicated significant differences when compared to the H_2_O_2_ treated group.

Although the free radical scavenging assays proved the excellent antioxidant activity of PSP-SeNPs, the antioxidant assays based on chemical reactions may not necessarily reflect the behavior of antioxidants in biological systems [16]. Thus, the effect of different selenium species on oxidative stress-induced damage to PC-12 cells was further investigated by MTT assay. As depicted in Figure 7A, the cell viability was higher than 90% when incubated with SeNPs and PSP-SeNPs at the concentration of 1–20 μg/mL. However, the cell viability dramatically decreased to 67% after treatment with 20 μg/mL Na_2_SeO_3_, suggesting that both SeNPs and PSP-SeNPs showed lower cytotoxicity than Na_2_SeO_3_.

The overproduction of reactive oxygen species (ROS) is considered to be the main cause of oxidative damage [44]. Herein, exogenous H_2_O_2_ was used as an inducer of cell damage in our model. PC-12 cells incubated with 500 μM H_2_O_2_ showed a remarkable decrease of cell viability reaching 56% (Figure 7B). However, the viability of PC-12 cells decreased to 55%, 50%, and 43% when pretreated with Na_2_SeO_3_ at concentrations of 1, 10, and 20 μg/mL, respectively. Interestingly, compared with the H_2_O_2_-induced oxidative stress model group, cells pretreated with SeNPs or PSP-SeNPs alleviated the H_2_O_2_-induced toxicity on PC-12 cells in a concentration-dependent manner, as reflected by the increase in cell viability. The viability of PC-12 cells pretreated with 20 μg/mL SeNPs or PSP-SeNPs significantly increased to 79% and 98%, respectively. In addition, the protective effect of PSP-SeNPs on H_2_O_2_-induced oxidative damage on PC-12 cells was better than that of SeNPs. The results confirmed that PSP-SeNPs had excellent antioxidant activity in cells, which may be associated with the free radical scavenging ability.

## 4. Conclusions

Our present study provided a facile approach for the synthesis of size-controlled SeNPs by using PSP as a stabilizer in the redox system of sodium selenite and ascorbic acid. The synthesized PSP-SeNPs presented a monodisperse spherical structure with zero-valent Se. The interaction between the hydroxyl groups of PSP chains and the surface of SeNPs contributed to the stable structure of PSP-SeNPs. Furthermore, PSP-SeNPs exhibited stronger free radical scavenging ability and a higher protective effect against H_2_O_2_-induced PC-12 cell death than SeNPs. Our findings not only provide the foundations for the utilization of PSP in the development of stable SeNPs but also emphasize the potential application of PSP-SeNPs as an antioxidant in food additives, dietary supplements, and nutraceuticals.

## Data Availability

The data presented in this study are available in this manuscript.
